# Comment on “Psychosocial work conditions and traffic safety among minibus and long-bus drivers”

**DOI:** 10.1093/joccuh/uiae038

**Published:** 2024-07-16

**Authors:** Sultan Pinar Cetintepe

**Affiliations:** Faculty of Medicine, Department of Public Health, Division of Occupational Medicine, Gazi University, 06560, Ankara, Turkey

**Keywords:** psychosocial work factors, road traffic safety, bus drivers, occupational health, Ghana

Dear Editor,

I am writing to provide feedback on the article “Psychosocial work conditions and traffic safety among minibus and long-bus drivers,” recently published in the *Journal of Occupational Health*. This study offers significant insights into the impact of psychosocial work factors on road traffic crashes (RTCs) among commercial bus drivers in Ghana. However, several critical areas could be explored further to enhance the discussion and provide a more comprehensive understanding.

Firstly, the study provides demographic data such as age and education levels but lacks a detailed analysis of how these demographics interact with psychosocial work factors.^1^ Understanding how age and education influence the perception and impact of psychosocial stressors could offer more targeted interventions. The differences between minibus and long-bus drivers regarding support and job demands are noted, but the cultural and social dynamics influencing these differences are not deeply explored.^1^^,^^2^ Understanding how cultural norms and organizational cultures within Ghana impact drivers' experiences and perceptions of psychosocial factors would provide valuable context.^1^

Secondly, economic pressures and their direct influence on driving behavior and RTCs are mentioned but not thoroughly analyzed.^3^ A deeper exploration of how financial incentives, pressures, and job insecurity drive risky behaviors and increased RTCs would be beneficial.^2^^,^^3^ This could include an analysis of income levels, economic dependency on moving jobs, and the financial stressors drivers face.^4^ Although the article concludes with general recommendations for improving occupational health and safety standards, more specific, actionable policy recommendations tailored to the Ghanaian context would be impactful.^1^ Detailed examples of successful interventions from similar contexts or pilot programs within Ghana could provide a roadmap for stakeholders.^1^^,^^4^ The cross-sectional design of the study limits the ability to draw causal inferences. Discussing the potential benefits of longitudinal studies to track changes in psychosocial work conditions and RTCs over time would be valuable. This approach could help in understanding the long-term effects of interventions and policy changes.^5^

Furthermore, the study does not address potential gender differences in psychosocial work conditions and RTCs.^5^ Given that gender dynamics can significantly impact occupational experiences and stress, an analysis of how male and female drivers might be differently affected and what tailored interventions might be necessary could provide a more nuanced understanding.^5^ In conclusion, although the study provides a solid foundation for understanding the relationship between psychosocial work conditions and traffic safety among bus drivers in Ghana,^1^ addressing these missing points could significantly enhance its impact and applicability. I look forward to future research that builds on these findings and offers more detailed solutions to the challenges faced by the occupational health community.
